# Comprehensive Analysis Reveals the Potential Diagnostic Value of Biomarkers Associated With Aging and Circadian Rhythm in Knee Osteoarthritis

**DOI:** 10.1111/os.14370

**Published:** 2025-01-23

**Authors:** Hao Li, Yuze Yang, Bo Li, Jiaju Yang, Pengyu Liu, Yuanpeng Gao, Min Zhang, Guangzhi Ning

**Affiliations:** ^1^ Department of Orthopedics Tianjin Medical University General Hospital, International Science and Technology Cooperation Base of Spinal Cord Injury, Tianjin Key Laboratory of Spine and Spinal Cord Tianjin China; ^2^ Department of Orthopedics The Second Hospital of Shanxi Medical University, Shanxi Key Laboratory of Bone and Soft Tissue Injury Repair Taiyuan China

**Keywords:** aging, circadian rhythm, DDIT4, knee osteoarthritis, mendelian randomization, PFKFB4

## Abstract

**Objective:**

Knee osteoarthritis (KOA) is characterized by structural changes. Aging is a major risk factor for KOA. Therefore, the objective of this study was to examine the role of genes related to aging and circadian rhythms in KOA.

**Methods:**

This study identified differentially expressed genes (DEGs) by comparing gene expression levels between normal and KOA samples from the GEO database. Subsequently, we intersected the DEGs with aging‐related circadian rhythm genes to obtain a set of aging‐associated circadian rhythm genes differentially expressed in KOA. Next, we conducted Mendelian randomization (MR) analysis, using the differentially expressed aging‐related circadian rhythm genes in KOA as the exposure factors, their SNPs as instrumental variables, and KOA as the outcome event, to explore the causal relationship between these genes and KOA. We then performed Gene Set Enrichment Analysis (GSEA) to investigate the pathways associated with the selected biomarkers, conducted immune infiltration analysis, built a competing endogenous RNA (ceRNA) network, and performed molecular docking studies. Additionally, the findings and functional roles of the biomarkers were further validated through experiments on human cartilage tissue and cell models.

**Results:**

A total of 75 differentially expressed aging‐circadian rhythm related genes between the normal group and the KOA group were identified by difference analysis, primarily enriched in the circadian rhythm pathway. Two biomarkers (PFKFB4 and DDIT4) were screened by MR analysis. Then, immune infiltration analysis showed significant differences in three types of immune cells (resting dendritic cells, resting mast cells, and M2 macrophages), between the normal and KOA groups. Drug prediction and molecular docking results indicated stable binding of PFKFB4 to estradiol and bisphenol_A, while DDIT4 binds stably to nortriptyline and trimipramine. Finally, cell lines with stable expression of the biomarkers were established by lentiviral infection and resistance screening, Gene expression was significantly elevated in overexpressing cells of PFKFB4 and DDIT4 and reversed the proliferation and migration ability of cells after IL‐1*β* treatment.

**Conclusions:**

Two diagnostic and therapeutic biomarkers associated with aging‐circadian rhythm in KOA were identified. Functional analysis, molecular mechanism exploration, and experimental validation further elucidated their roles in KOA, offering novel perspectives for the prevention and treatment of the disease.

## Graphical abstract

This study employed transcriptomic analysis and Mendelian randomization to identify diagnostic and therapeutic biomarkers associated with aging‐circadian rhythm in knee osteoarthritis. Further functional and molecular mechanism analyses were conducted to elucidate their roles in knee osteoarthritis, providing a theoretical basis for clinical diagnosis and treatment of the disease.

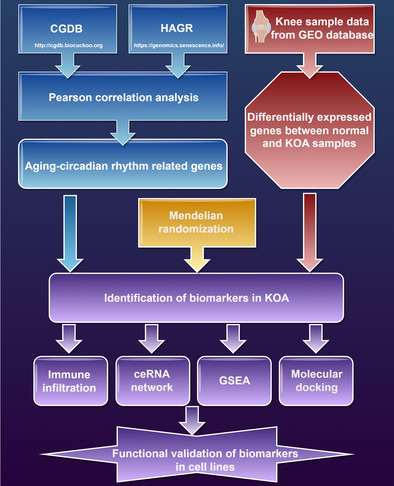

## 1 Introduction

Knee osteoarthritis (KOA) is common degenerative disease characterized primarily by articular cartilage degradation, subchondral bone loss, synovial inflammation, and joint instability. Clinically, it manifests as joint pain, swelling, and impaired mobility, ultimately leading to disability [1, 2, 3]. The prevalence of KOA is rising due to aging and increasing obesity, with estimates indicating over 500 million individuals globally affected by osteoarthritis, imposing substantial burdens on healthcare systems and significant economic strain on patients and society [4, 5, 6, 7]. Historically, clinical approaches to KOA have primarily focused on alleviating joint pain symptoms [8, 9]; however, recent strategies have increasingly emphasized early prevention [10, 11]. This shift aims to achieve better therapeutic outcomes by halting or delaying disease progression before extensive joint destruction occurs, highlighting the urgent need to identify potential biomarkers and therapeutic targets for KOA.

Aging is a complex biological process characterized by changes in gene expression, cellular function, and systemic decline, contributing to the onset of various chronic diseases [12, 13, 14]. Therefore, aging is also considered a significant risk factor for the onset and progression of KOA [15]. Circadian rhythm, defined as the 24‐h periodicity of physiological processes, is regulated by the endogenous biological clock [16]. The stability of circadian rhythm is critical for cellular, tissue, and organismal health. Recent studies have elucidated a significant interaction between aging and circadian rhythms [17, 18, 19]. As organisms age, circadian rhythms deteriorate, evidenced by a decline in the amplitude of sleep/wake cycles, body temperature, and plasma hormone levels [20]. Furthermore, disruptions in circadian rhythm functionality can lead to tissue degeneration and premature aging [20].

In KOA, circadian rhythm genes play a crucial role in regulating the biological rhythms of chondrocytes and synovial tissues, and aging may influence the expression of these genes by exacerbating inflammation and oxidative stress, thereby affecting cellular metabolism and function [21]. Conversely, circadian rhythm disruption could accelerate the aging process of chondrocytes [22]. This interaction between aging and circadian rhythm genes may establish a complex feedback loop that accelerates cartilage degradation and impairs joint repair.

KOA is currently incurable, and early diagnosis and early intervention are particularly important. However, the roles and mechanisms of aging and circadian rhythms in KOA remain incompletely understood. Thus, investigating the effects of aging‐circadian rhythms on KOA and identifying key aging‐associated circadian genes and their specific functions could enhance our understanding of KOA pathogenesis at the molecular level, providing a theoretical basis for identifying early biomarkers and novel therapeutic targets. The purposes of this study were to: (i) clarify the relationship between circadian genes and aging‐induced KOA, (ii) identify biomarkers of KOA and validate their roles, and (iii) comprehensively analyze the biomarkers of KOA.

## 2 Materials and Methods

### 2.1 Data Collection

Transcriptome data were obtained from the Gene Expression Omnibus (GEO) database (https://www.ncbi.nlm.nih.gov/geo). The training set (GSE114007) consisted of knee cartilage tissue from the knee joints from 18 normal and 20 osteoarthritis (OA) samples. These samples were sequenced using GPL11154 and GPL18573 sequencing platforms. The validation set (GSE51588) on sequencing platform of GPL13497, included 10 normal tissue samples and 40 subchondral bone tissue samples from the lateral and medial tibial plateaus of the knee joints of OA patients. A total of 1111 circadian rhythm‐related genes (CIRGs) were collected from the CGDB database (http://cgdb.biocuckoo.org). Additionally, 307 aging‐related genes (ARGs) were obtained from the HAGR database (https://genomics.senescence.info/) from GenAge, and 949 ARGs were collected from CellAge. After merging and removing duplicate genes, a total of 1061 ARGs were identified. The ebi‐a‐GCST007090 for KOA was obtained from the European Bioinformatics Institute. This dataset comprised 403,124 samples, including 378,169 control samples and 24,955 case samples, with a total of 29,999,696 single nucleotide polymorphisms (SNPs) and represented individuals of European ethnicity.

### 2.2 Screening of the Differentially Expressed Aging‐Circadian Rhythm Related Genes (DE‐ARGs_CIRGs)

The limma package [23] was utilized to compare the differentially expressed genes (DEGs) between the KOA and normal groups within the GSE114007 dataset, with filtering conditions set at |log_2_FC| > 1 and P adjust < 0.05. The ggplot2 and pheatmap packages [24] were employed to generate volcano plots and heat maps, respectively, illustrating the differential expression of genes. Additionally, principal component analysis (PCA) plots of KOA and normal samples were generated using the factoextra package [25].

Based on the collected ARGs and CIRGs, their Pearson correlations were analyzed. A threshold of |*R*| > 0.3, *p* < 0.05 was used to screen out aging‐circadian rhythm related genes (ARGs_CIRGs) with a correlation greater than 30% of the total, and a correlation heat map was generated. The intersection of ARGs_CIRGs and DEGs was obtained to identify the DE‐ARGs_CIRGs in KOA and a Venn diagram was generated using the ggvenn package [26] for visualization purposes.

### 2.3 Functional Enrichment Analysis and Gene Set Variation Analysis (GSVA)

The clusterProfiler package [27] was utilized to conduct enrichment analysis of DE‐ARGs_CIRGs in KOA. The enrichGO function in the clusterProfiler package was employed for Gene Ontology (GO) enrichment analysis, and the GOplot package [28] was used to generate network diagrams for visualizing the association between the most significant GO terms and genes. Additionally, the enrichKEGG function in the clusterProfiler package was applied for Kyoto Encyclopedia of Genes and Genomes (KEGG) enrichment analysis. A *p* adjust value < 0.05 served as the threshold for enrichment analysis.

Based on the “Hallmark” background gene set (Hallmark.all.v2022.1.Hs.symbols) and the “KEGG” background gene set (c2.cp.kegg.symbols) from the MSigDB database (https://www.gsea‐msigdb.org/gsea/msigdb), we employed the GSVA [29] and limma packages for GSVA enrichment analysis. For comparisons between the KOA group and the normal group, individual entries were analyzed for differences using the limma package, applying significance thresholds of |*t*| > 2 and *p* value < 0.05. To evaluate enrichment in cartilage samples from normal versus KOA conditions, we scored Hallmark and KEGG pathways utilizing the GSVA package, with an FDR threshold of < 0.05 established as a criterion for significant enrichment.

### 2.4 Screening Candidate Genes by MR Analysis

In this study, we utilized the DE‐ARGs_CIRGs as exposure factors and KOA as the outcome event for MR analysis. Genes that have a causal relationship with KOA were identified as candidate genes. The assumptions underlying the MR analyses are as follows: (1) SNPs serve as instrumental variables (IVs) for exposure; (2) these IVs are not influenced by confounding factors; and (3) the risk of the outcome is affected solely through the exposure pathway.

We analyzed the eQTL of the genes based on a threshold of *p* < 5E‐06, excluding target genes with less than 3 IVs and removing IVs with linkage disequilibrium using parameter settings: *r* ^2^ = 0.001; kb = 100. Additionally, weak IVs with an *F*‐statistic value less than 10 were also removed. Confounders were excluded through the GWAS Catalog database (https://www.ebi.ac.uk/gwas/), by excluding SNPs potentially associated with the ending GWAS trait using a threshold of *p* < 1E‐05. Effect alleles and effect sizes were harmonized using the TwoSampleMR package [30] function harmonise_data, and effect sizes of IVs significantly associated with the outcome were excluded and combined with five algorithms (MR Egger, Weighted median, Inverse variance weighted (IVW), Simple mode, and Weighted mode) to perform MR analyses for the IVW decisive approach.

Sensitivity analyses were conducted on the results of the MR analyses. The Cochran *Q* test was employed to evaluate the heterogeneity among SNPs, while the MR‐Egger intercept was utilized to detect horizontal pleiotropic effects. A *p* value greater than 0.05 served as an indicator for the absence of heterogeneity and multiple effects. Furthermore, leave‐one‐out analyses were performed to identify SNPs that may exert extreme influences on the estimates, thereby enhancing the reliability of the findings.

### 2.5 Identification of Biomarkers and Chromosomal Localization Analyses

To evaluate the diagnostic potential of the selected candidate genes, we generated receiver operating characteristic (ROC) curves for GSE114007 and GSE51588 datasets and the pROC package [31] was utilized to calculate the area under the curve (AUC) values. This approach enabled us to identify candidate genes with robust diagnostic capabilities for further analysis. Additionally, we constructed box plots to illustrate the expression levels of these candidate genes across both datasets, allowing us to examine biomarker expression and select those candidates exhibiting consistent expression trends as potential biomarkers. Furthermore, chromosomal position analysis was carried out on the identified biomarkers employing the RCircos package [32] to ascertain their genomic locations on chromosomes.

### 2.6 Gene Set Enrichment Analysis (GSEA) of Biomarkers

In the GSE114007 dataset, a Spearman correlation analysis was conducted between each biomarker and all genes utilizing the psych package [33]. Based on the ranking of the correlation results, GSEA pathway enrichment analysis was performed using the clusterProfiler package. The gene set files required for GSEA pathway enrichment can be downloaded from the official GSEA website (http://www.gsea‐MSigdb.org/gsea/msigdb).

### 2.7 Immune Infiltration Analysis

Based on GSE114007, immune infiltration analysis was performed. The number of immune cells infiltrated was assessed by the CIBERSORT package [34]. The ggplot2 package presented the abundance and proportion of immune infiltration for each sample. The Wilcoxon test was used to compare the difference in the proportions of the 22 immune cells in the KOA samples and the control samples. In addition, Pearson correlation coefficients among 22 types of immune cells were computed using the corrplot package [35] to assess the relationships between various immune cell types. Furthermore, Spearman correlation coefficients between biomarkers and the 22 types of immune cells were determined utilizing the ggstatsplot package [36].

### 2.8 Construction of Competitive Endogenous RNA (ceRNA) Network

Based on the identified biomarkers, microRNAs (miRNAs) were predicted using the miRDB (https://mirdb.org/) database and StarBase (https://rnasysu.com/encori/) database. The prediction of miRNAs was followed by an analysis of miRNA and long non‐coding RNAs (lncRNAs) interactions in the StarBase database and the LncBase Predicted v.2 database. Subsequently, the ceRNA network was constructed using Cytoscape.

### 2.9 Drug Prediction and Molecular Docking

Biomarkers were inputted into the DGIdb database (https://www.dgidb.org/) for the prediction of potential candidate drug. The candidate drugs were then retrieved from the PubChem database (https://pubchem.ncbi.nlm.nih.gov/) to obtain their 3D structures. Additionally, the biomarkers were imported into the UniProt and PDB to acquire receptor structures at the highest resolution. Dehydration, hydrogenation, and charge setting were performed using AutoDock and PyMOL software. Molecular docking was conducted with AutoDock software to explore biomarker‐drug binding modes.

### 2.10 Clinical Samples

To validate the expression of core genes, we collected 13 human knee cartilage specimens during our clinical work. Among these, 8 were osteoarthritic cartilage samples from patients who underwent total knee arthroplasty due to KOA, while 5 were normal cartilage tissues from patients who underwent amputation for non‐KOA conditions. Detailed information regarding the cartilage donors is provided in Table S1. This study was approved by the Ethics Committee of the Second Hospital of Shanxi Medical University, and all participants provided informed consent by reading and signing the consent form.

### 2.11 Quantitative Real‐Time PCR (RT‐qPCR)

Total RNA was extracted using TRIzol reagent (Invitrogen, USA) from cartilage tissue and cultured cells. Complementary DNA was synthesized with a reverse transcription kit (Takara, Japan). RT‐qPCR was performed with SYBR Green reagent via the ABI7500 system (Applied Biosystems, USA). The relative gene expression was calculated by 2^−ΔΔCT^ method, and β‐actin was used as internal reference to standard expression level. The sequences of primers used were listed in Table S2.

### 2.12 Cell Culture and Treatment

ATDC5 cells cultured in DMEM containing nutrient mixture F12 supplemented with 10% fetal bovine serum in a humidified atmosphere with 5% CO_2_ at 37°C. Cell lines stably expressing DDIT4 and PFKFB4 were established through lentiviral infection and resistance screening. Both ATDC5 and the two stably transduced cell lines were cultured overnight, after which 10 ng/mL IL‐1β was added to induce cellular inflammation. The experimental groups were designated as follows: IL‐1β, IL‐1β + DDIT4^OE^ (IL‐1β‐treated DDIT4 stably expressing cell line), and IL‐1β + PFKFB4^OE^ (IL‐1β‐treated PFKFB4 stably expressing cell line).

### 2.13 Cell Counting Kit‐8 (CCK‐8)

Cell proliferation was assessed using the CCK‐8 assay (Keygen Biotech Co. Ltd., China). Cells were plated in 96‐well plates at a density of 4 × 10^3^ cells/well and treated with IL‐1β for 24, 48, and 72 h. Following treatment, 10 μL of CCK‐8 reagent was added to each well, and the cells were incubated at 37°C for 2 h. Finally, the OD values were measured at 450 nm with a microplate reader.

### 2.14 The Experiment of 5‐Ethy‐ nyl‐2′‐Deoxyuridine (EDU)

EDU kit (RiboBio, China) was used to further evaluate cell proliferation. Cells were inoculated in 24‐well plates at a density of 3.0 × 10^4^ cells/well, treated with IL‐1β for 24 h, and then added with EDU solution for co‐incubation for 2 h. Then, Apollo reaction mixture was added to each well and incubation was continued in the dark for 30 min. Subsequently, the nuclei of the cells were stained with Hoechst 33258 for 30 min. Finally, cell proliferation images were taken using fluorescence microscopy and the number of EDU‐positive cells was quantified by ImageJ software.

### 2.15 Cell Scratch Assay

The scratch healing assay was employed to assess cell migration capacity. Cells were seeded in 6‐well plates at a density of 1 × 10^6^ cells/well and allowed to reach 100% confluence overnight. Scratches were made on the cell surface using a sterile 200 μL tip. The medium was then replaced with serum‐free medium containing IL‐1β, and the cells were incubated for 12 h. Cell images were taken at 0 and 12 h using a light microscope, and the scratch healing ability was analyzed using ImageJ software.

### 2.16 Transwell Migration Assay

Transwell chambers (Corning, USA; pore size: 8 μm) were employed for the assessment of cell migration. Cells were resuspended in complete medium containing 1% FBS and inoculated into the upper chamber of 24‐well plates at a density of 3 × 10^4^ cells per well. IL‐1β was introduced to the upper chamber, while 750 μL of complete medium supplemented with 10% FBS was added to the lower chamber. The cells were incubated for a duration of 24 h. Following incubation, cells adhering to the upper surface of the filter membrane were removed using a cotton swab, and those on the lower surface were stained with a solution of 0.5% crystal violet for 30 min. The number of migrated cells was subsequently observed under a light microscope and quantified utilizing ImageJ software.

### 2.17 Statistical Analysis

All analyses were conducted using R packages (version 4.2.1). For significance testing, either the Wilcoxon test or t‐test was employed to compare differences between the two sample groups. In the experimental validation section, all data are expressed as mean ± standard deviation. All experiments were performed at least three times, and data were statistically analyzed and visualized using GraphPad Prism software. Differences between two groups were assessed using a *t*‐test. Two‐sided tests were considered statistically significant at a *p* value < 0.05.

## 3 Results

### 3.1 Identification of DE‐ARGs_CIRGs in KOA

**FIGURE 1:**
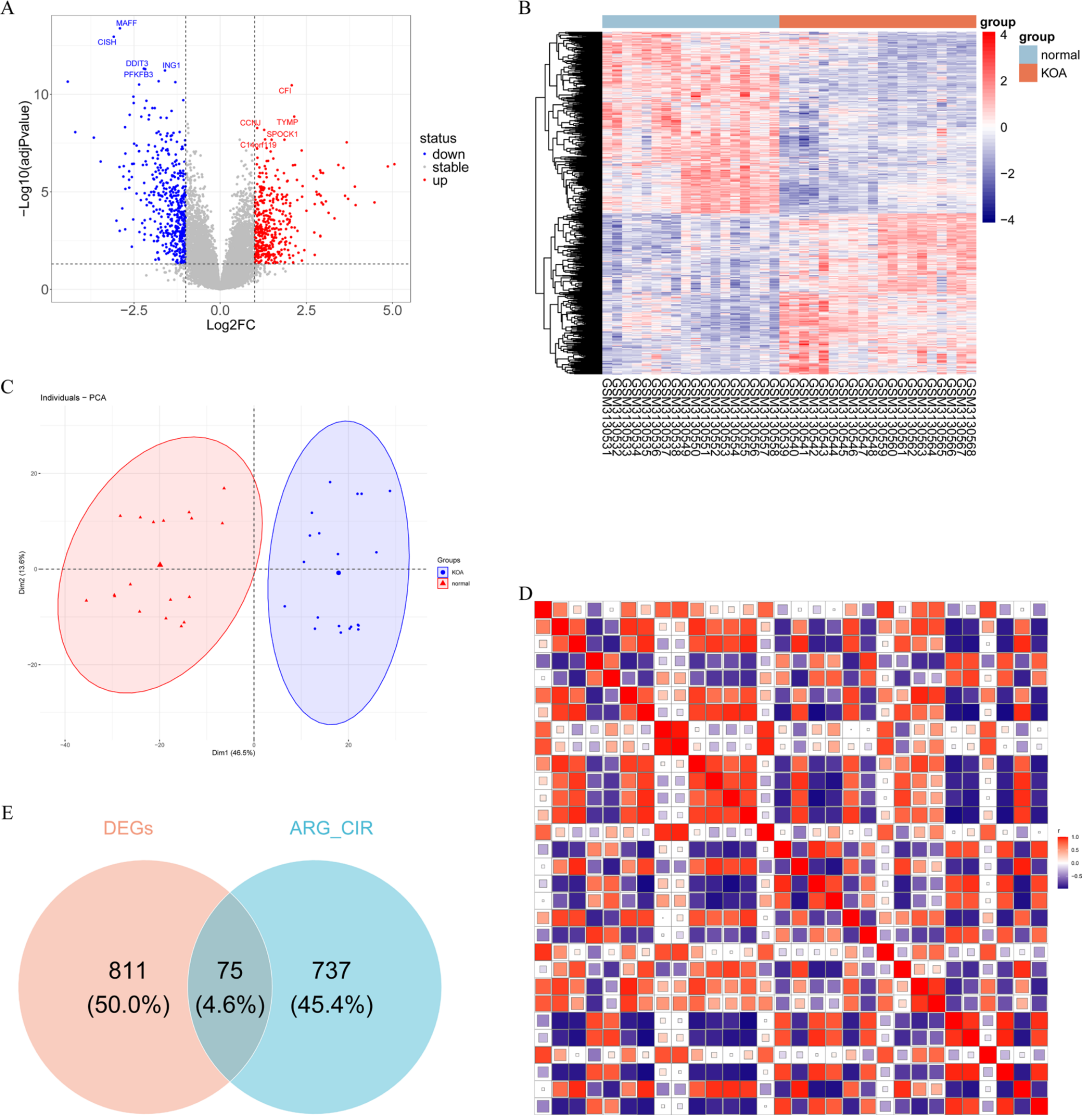
Screening of the differentially expressed aging‐circadian rhythm related genes (DE‐ARGs_CIRGs) in knee osteoarthritis (KOA). (A) Volcano map of differentially expressed genes (DEGs) in the GSE114007 dataset. (B) Heatmap illustrated the expression levels of DEGs. Genes with high expression are represented in red, while those with low expression are depicted in blue. (C) Principal component analysis (PCA) analysis of normal and KOA samples. (D) Heatmap of correlations between 30 aging‐related genes and 30 circadian rhythm‐related genes. (E) Venn diagram of DEGs and aging‐related_circadian rhythm‐related genes.

A total of 886 DEGs were identified through differential analysis, with 416 being up‐regulated and 470 being down‐regulated (Figure 1A). The heatmap visually represented the expression patterns of DEGs in both normal and KOA samples (Figure 1B). PCA revealed that principal component 1 accounted for 46.5% of the variance, while principal component 2 accounted for 13.6% (Figure 1C). The scatter plot showed that the normal and KOA samples formed distinct clusters within their respective groups, indicating high reproducibility within each group. Furthermore, there was clear differentiation between the two groups, suggesting a significant difference in gene expression between the KOA group and the normal group (Figure 1D).

Based on ARGs and CIRGs, a total of 812 ARGs_CIRGs were identified using the Pearson correlation method. The heatmap illustrated the correlation between 30 ARGs and 30 CIRGs. The intersection of ARGs_CIRGs and DEGs yielded a set of 75 DE‐ARGs_CIRGs in KOA (Figure 1E).

### 3.2 Exploring the Potential Function of DE‐ARGs_CIRGs and KOA

**FIGURE 2:**
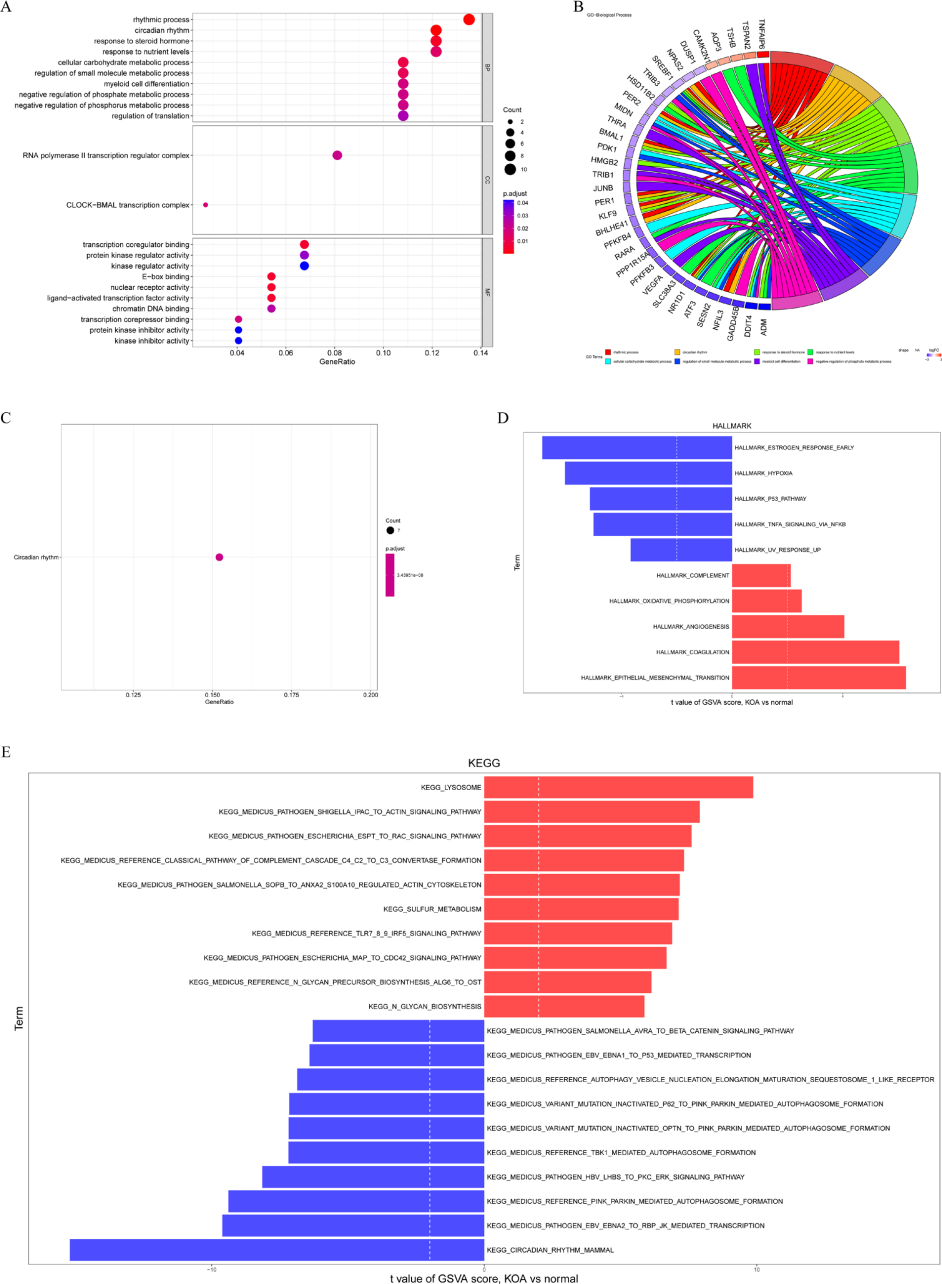
Functional enrichment analysis and Gene Set Variation Analysis (GSVA). (A) Bubble chart of Gene Ontology (GO) enrichment analysis. BP, biological process; CC, cellular component; MF, molecular function. (B) The circos diagram of GO enrichment analysis. (C) Bubble chart of Kyoto Encyclopedia of Genes and Genomes (KEGG) enrichment analysis. (D) GSVA in “Hallmark” background. (E) GSVA in “KEGG” background.

To elucidate the molecular function and mechanism of action of DE‐ARGs_CIRGs in KOA, an enrichment analysis was conducted. The GO enrichment analysis revealed DE‐ARGs_CIRGs were significantly associated with circadian rhythm, regulation of translation, the CLOCK‐BMAL transcription complex, and transcriptional regulation. A network diagram was constructed to visualize the relationships among the most prominent GO terms and their corresponding genes (Figure 2A,B). In addition, KEGG pathway enrichment analysis identified a single enriched pathway related to circadian rhythm (Figure 2C). These findings suggest that the circadian rhythm pathway may play a role in disease progression.

To gain a deeper understanding of the pathways involved in KOA, we conducted GSVA enrichment analysis. The GSVA enrichment analysis utilizing the “Hallmark” background gene set indicated that the primary pathways associated with KOA include hypoxia, the P53 pathway, and epithelial‐mesenchymal transition (Figure 2D). Furthermore, the GSVA enrichment analysis based on the “KEGG” background gene set identified lysosome‐related processes, circadian rhythm in mammals, and autophagy as significant pathways implicated in KOA (Figure 2E).

### 3.3 Identification of Candidate Genes With Causal Relationship to KOA

**FIGURE 3:**
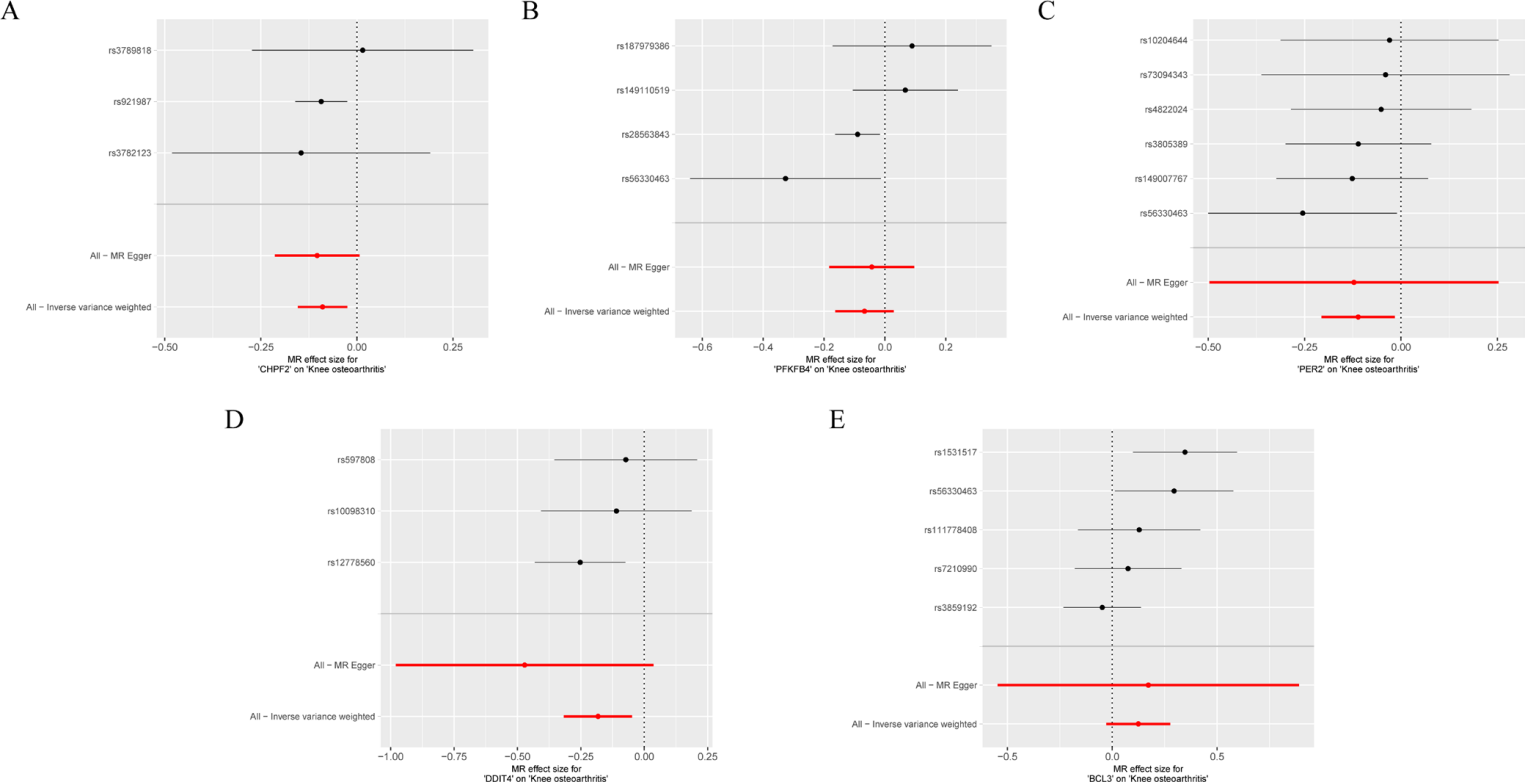
Forest plots combined with individual single nucleotide polymorphism (SNP) estimates risk effects related to biomarkers and KOA. (A) The forest map illustrated the causal effects of SNPs associated with CHPF2 on KOA. (B) The forest map illustrated the causal effects of SNPs associated with PFKFB4 on KOA. (C) The forest map illustrated the causal effects of SNPs associated with PER2 on KOA. (D) The forest map illustrated the causal effects of SNPs associated with DDIT4 on KOA. (E) The forest map illustrated the causal effects of SNPs associated with BCL3 on KOA.

To investigate the causal relationship between DE‐ARGs_CIRGs and KOA, a MR analysis was conducted. Genes demonstrating a significant causal association with KOA were identified as candidate genes. After conducting a series of screenings, a total of 59 genes were ultimately retained for further analysis (Table S3). The IVW results indicated that CHPF2 (odds ratio (OR) = 0.914, *p* = 0.006), BCL3 (OR = 1.132, *p* = 0.024), PFKFB4 (OR = 0.935, *p* = 0.041), PER2 (OR = 0.895, *p* = 0.022), and DDIT4 (OR = 0.834, *p* = 0.008) exhibited significant causal relationships with the outcome of KOA. It indicated that CHPF2 (Figure 3A), PFKFB4 (Figure 3B), PER2 (Figure 3C), and DDIT4 (Figure 3D) functioned as protective factors for KOA, while BCL3 (Figure 3E) was identified as a risk factor for KOA (Table 1).

**TABLE 1:** Mendelian randomization results of differentially expressed aging‐circadian rhythm related genes and knee osteoarthritis.

Outcome	Exposure	Method	nsnp	*b*	SE	*p*	or	or_lci95	or_uci95	pleio_P	heter_P
Knee osteoarthritis	CHPF2	Inverse variance weighted	3	−0.089	0.033	0.006	0.914	0.857	0.975	0.810	0.732
	BCL3		5	0.124	0.055	0.024	1.132	1.016	1.262	0.901	0.093
	PFKFB4		4	−0.067	0.033	0.041	0.935	0.877	0.997	0.641	0.082
	PER2		6	−0.111	0.048	0.022	0.895	0.814	0.984	0.956	0.834
	DDIT4		3	−0.182	0.069	0.008	0.834	0.729	0.954	0.453	0.494

Abbreviations: b, beta; ci, confidence interval; heter_P, heterogeneity significance; nsnp, number of single nucleotide polymorphisms used for analysis; or, odds ratio; pavl, significance; pleio_P, horizontal pleiotropy significance; se, standard error.

**FIGURE 4:**
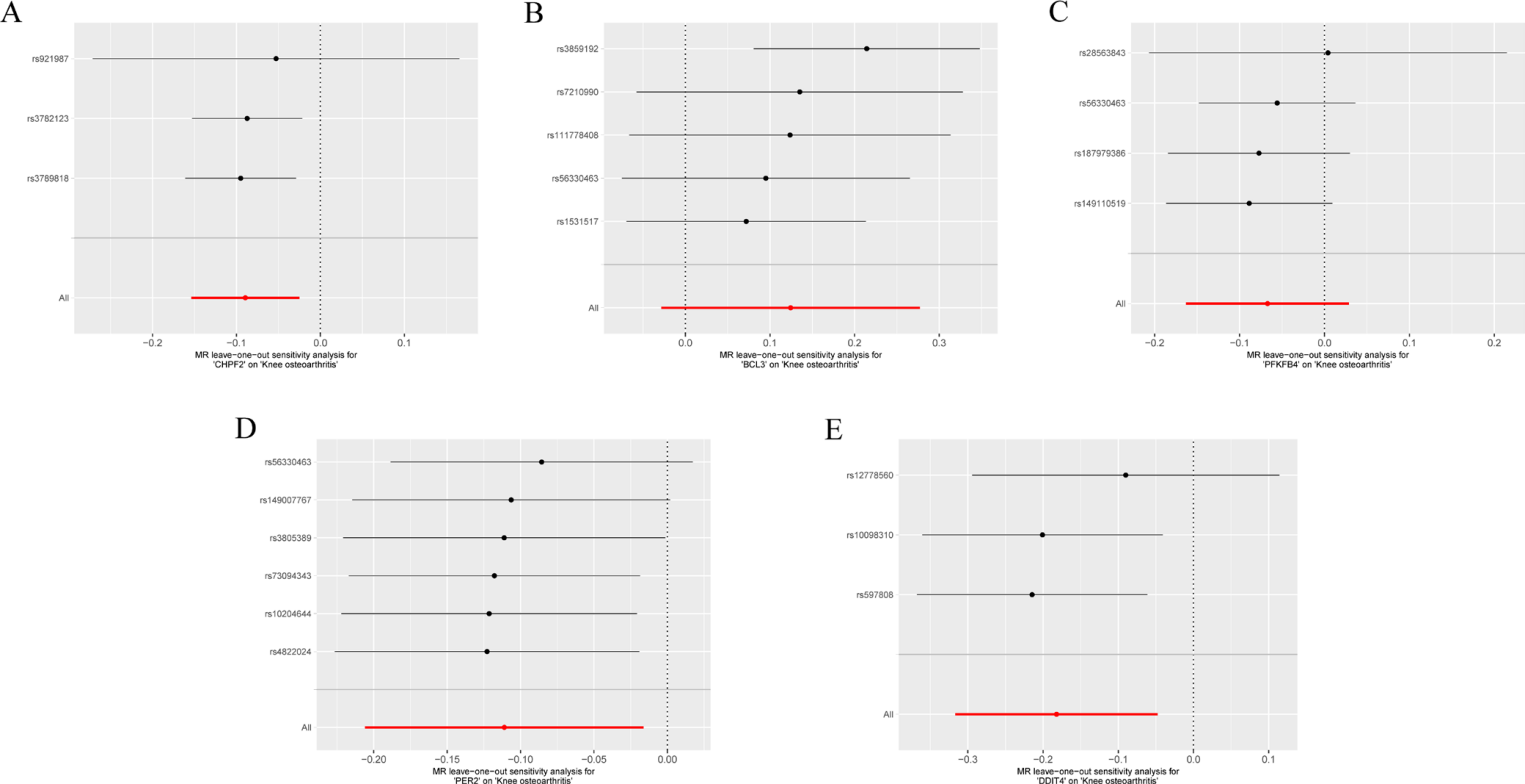
Leave‐one‐out test for Mendelian randomization (MR) of biomarkers. (A–E) Leave‐one‐out forest map for CHPF2, BCL3, PFKFB4, PER2, and DDIT4 on KOA, respectively. Each line in the figure represented the effect of the model when that specific line of SNPs was excluded.

In order to evaluate the reliability of the results from the MR analysis, a sensitivity analysis was performed. The heterogeneity test results for CHPF2, BCL3, PFKFB4, PER2, and DDIT4 indicated no significant heterogeneity (Table 1, Figure S1). The *p* values associated with horizontal pleiotropy for these variables were all greater than 0.05, suggesting that horizontal pleiotropy effects were not observed (Table 1); thus, there were no confounding factors in this study. This further enhances the reliability of our findings. Additionally, by systematically removing each SNP from CHPF2 (Figure 4A), BCL3 (Figure 4B), PFKFB4 (Figure 4C), PER2 (Figure 4D), and DDIT4 (Figure 4E) individually, we found that the effects of the remaining SNPs on the outcome variables did not change significantly. This observation indicates that the results of the MR analysis demonstrate strong reliability and stability.

### 3.4 Acquisition, Chromosomes Localization and Validation of Biomarkers

**FIGURE 5:**
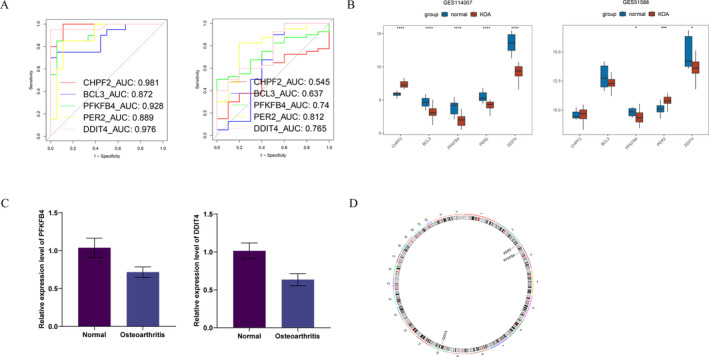
Diagnostic ability assessment, expression level verification and chromosome localization of biomarkers. (A) Receiver operating characteristic (ROC) curves for biomarkers in training set GSE114007 and validation set GSE51588, respectively. (B) Expression level box plots for biomarkers in training set GSE114007 and validation set GSE51588 between KOA and normal groups, respectively. (C) Differential mRNA expression of PFKFB4 and DDIT4 in normal cartilage tissues and osteoarthritis cartilage tissues. (D) The circle diagram of biomarkers localization on chromosomes.

To further evaluate the biomarkers, we initially plotted ROC curves. The results indicated that PFKFB4, PER2, and DDIT4 exhibited AUC values exceeding 0.7 in both the training and validation sets, demonstrating their strong diagnostic potential (Figure 5A); thus, these three were selected for subsequent analysis. Subsequently, we generated box plots of expression levels and observed that PFKFB4, PER2, and DDIT4 displayed significant differences across both datasets, which aligned with the findings from the ROC analysis. However, it is noteworthy that the trend of PER2 expression was inconsistent between the two datasets (Figure 5B). Consequently, we ultimately selected PFKFB4 and DDIT4 as biomarkers for further investigation. The RT‐qPCR further confirmed that PFKFB4 and DDIT4 expression at the mRNA level was lower in osteoarthritis cartilage tissue than in normal cartilage tissues (Figure 5C). These results were consistent with the results of the bioinformatics analysis. It was further evidence that the genes we screened for may serve as diagnostic biomarkers for KOA. In addition, we conducted an analysis of the biomarkers concerning their chromosomal locations. The findings indicated that PFKFB4 was situated on chromosome 3, whereas DDIT4 was localized to chromosome 10 (Figure 5D).

### 3.5 The Functions and Regulatory of Biomarkers

**FIGURE 6:**
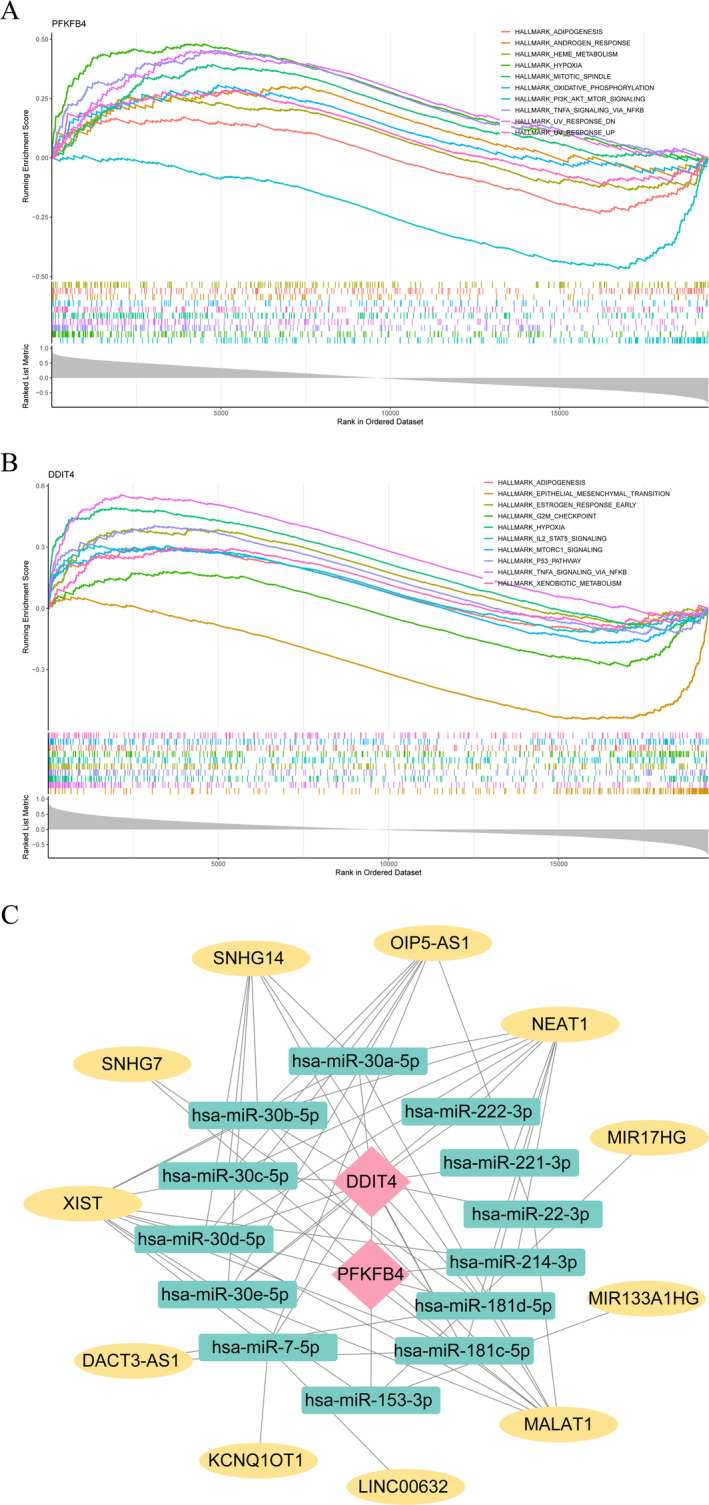
Gene Set Enrichment Analysis (GSEA) and competing endogenous RNA (ceRNA) network. (A) GSEA of PFKFB4. (B) GSEA of DDIT4. (C) ceRNA of biomarkers. Pink denoted biomarkers, green signified microRNAs (miRNAs), and yellow indicated long non‐coding RNAs (lncRNAs).

To investigate the functional roles of the biomarkers, GSEA analysis was conducted. The results indicated that DDIT4 and PFKFB4 were significantly enriched in both hypoxia and TNF signaling pathways via NFKB. Furthermore, DDIT4 showed predominant enrichment in epithelial‐mesenchymal transition (EMT) and the P53 pathway, whereas PFKFB4 exhibited primary enrichment in mitotic spindle formation and oxidative phosphorylation (Figure 6A,B).

In order to gain a deeper understanding of the interactions within the gene regulatory network, we conducted a construction of the ceRNA network. The results indicated that 13 miRNAs targeting PFKFB4 and DDIT4 were identified. Furthermore, we predicted the interactions between these 13 miRNAs and 11 lncRNAs, leading to the establishment of the ceRNA network. This network comprised a total of 26 nodes and 63 interaction pairs. Our analysis revealed that hsa‐miR‐30c‐5p and hsa‐miR‐30d‐5p are capable of regulating both PFKFB4 and DDIT4. Additionally, XIST may influence the expression levels of PFKFB4 and DDIT4 by indirectly modulating hsa‐miR‐30d‐5p and hsa‐miR‐30c‐5p, respectively (Figure 6C).

### 3.6 Discrepancies of Immune Microenvironment Between KOA and Normal Groups

**FIGURE 7:**
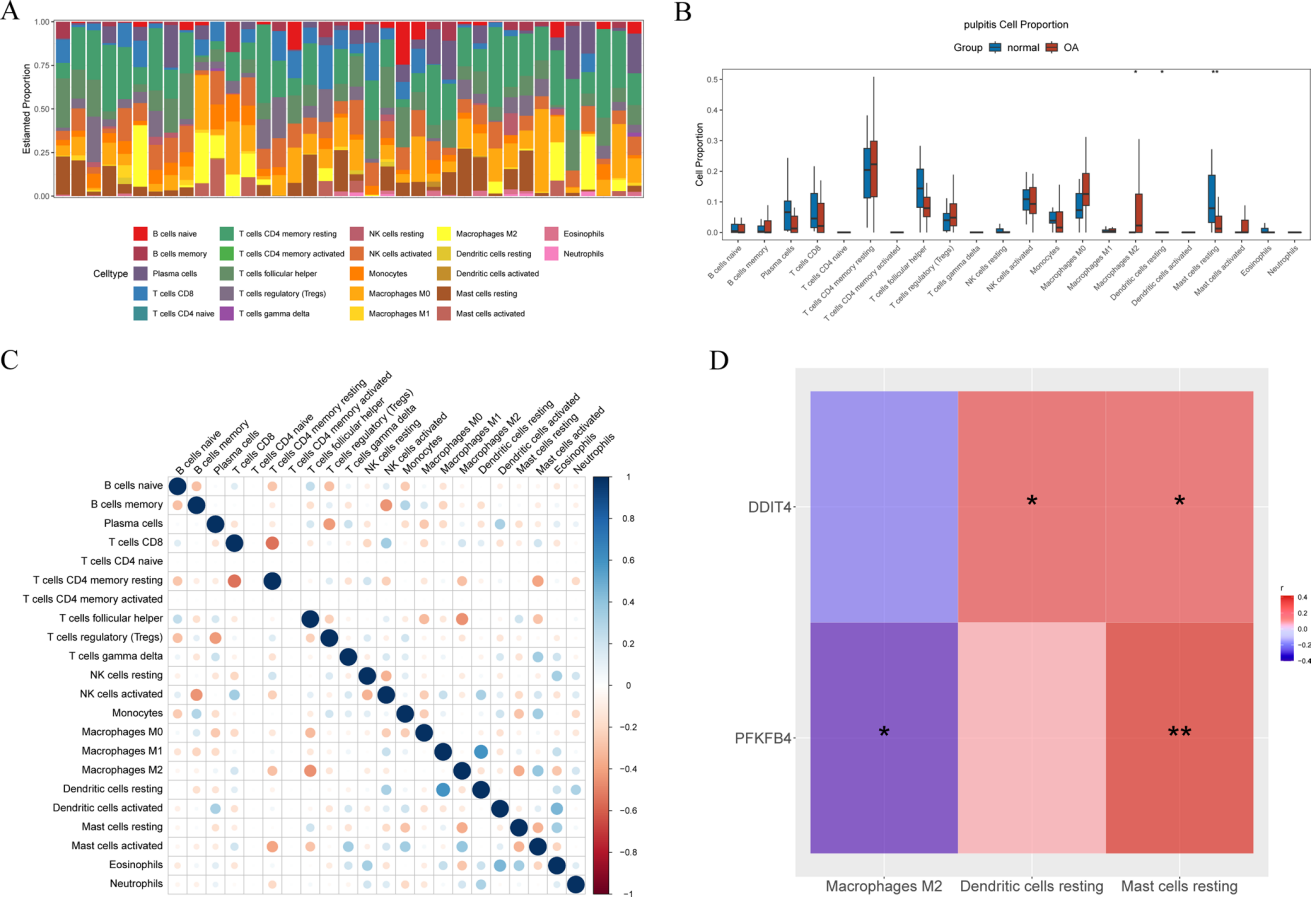
Immune infiltration analysis. (A) Stack bar diagram of 22 immune cells score. (B) Box plot of 22 immune cells proportion between different groups in normal and KOA groups. “*” represents *p* < 0.05, “**” represents *p* < 0.01. (C) Pearson correlation heat map between immune cells. (D) Heat map of correlation between biomarkers and differential immune cells. “*” represents *p* < 0.05, “**” represents *p* < 0.01.

To investigate the infiltration of immune cells, an immune infiltration analysis was conducted. Bar graph was generated to illustrate the percentage abundance of 22 different immune cell types in KOA samples compared to control samples (Figure 7A). Subsequently, a Wilcoxon test was performed to compare immune cell populations between disease and control groups, revealing significant differences in three specific types of immune cells: M2 macrophages, resting dendritic cells, and resting mast cells (Figure 7B). Furthermore, the correlation among various types of immune cells was analyzed, which indicated a positive correlation between resting dendritic cells and M1 macrophages (Figure 7C). To explore the relationship between biomarkers and differential immune cell populations, a correlation analysis was carried out. Notably, PFKFB4 exhibited the highest positive correlation with resting mast cells (correlation coefficient (*r*) = 0.416), while it demonstrated the strongest negative correlation with M2 macrophages (*r* = −0.401) (Figure 7D). The aforementioned findings indicated a modification in the immune microenvironment, which may influence the progression of KOA.

### 3.7 Prediction of Potential Drug Targets for KOA

**FIGURE 8:**
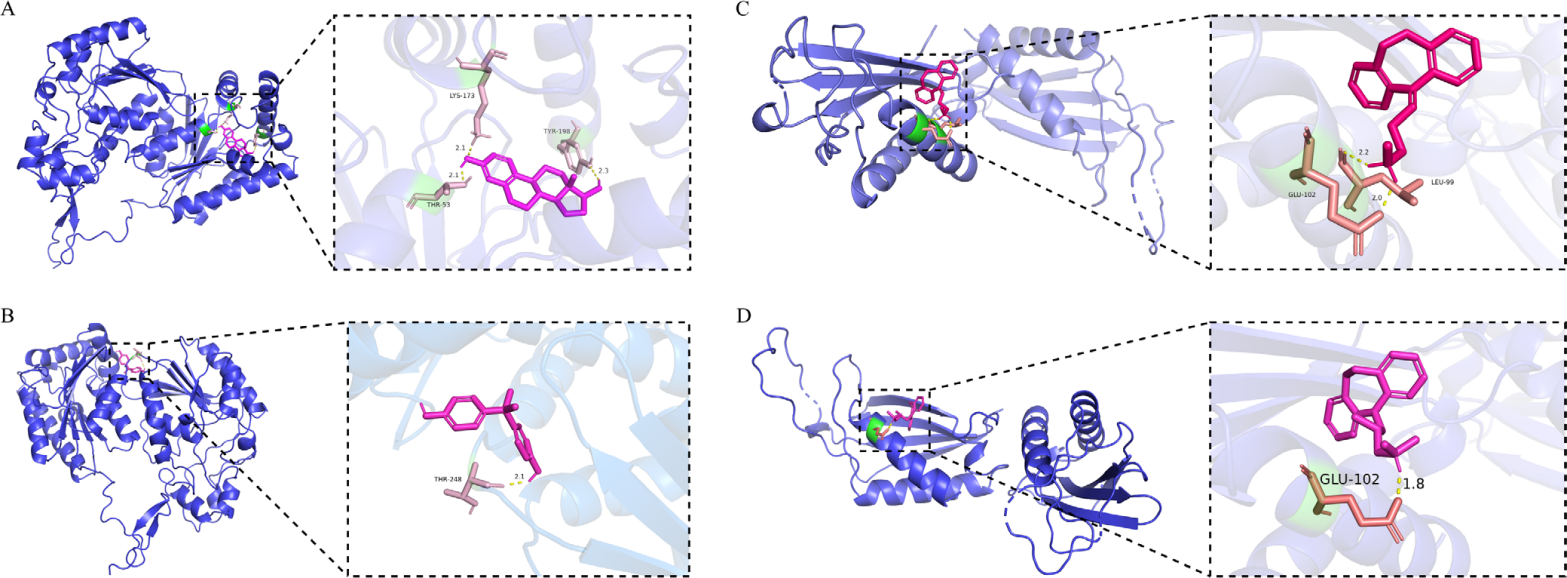
Results of molecular docking of biomarkers and drugs. (A) PFKFB4 and estradiol. (B) PFKFB4 and bisphenol_A. (C) DDIT4 and nortriptyline. (D) DDIT4 and trimipramine.

In order to explore potential therapeutic agents for KOA, the drug prediction analysis was conducted. The results indicated that 128 potential drugs targeting PFKFB4 (Table S4), while 7 targeting DDIT4 (Table 2). To evaluate the binding affinity of these drugs to specific biomarkers, two drugs that demonstrated relatively high scores were selected for molecular docking analysis. The results indicated that the binding energies of PFKFB4 with estradiol (−7.87 kcal/mol) and bisphenol A (−5.87 kcal/mol) were relatively low; similarly, the binding energies of DDIT4 with nortriptyline (−6.31 kcal/mol) and trimipramine (−6.13 kcal/mol) also exhibited low values (Figure 8A–D). These findings suggested that both PFKFB4 and DDIT4 can stably bind to relevant drugs, indicating their potential as therapeutic options for KOA.

**TABLE 2:** Drug prediction results of DDIT4.

Gene	Drug	Regulatory approval	Indication	Interaction score
DDIT4	Desipramine	Approved	Tricyclic, Antidepressive Agents	0.192
	Monoamine oxidase inhibitor	Not Approved		0.259
	Doxepin hydrochloride	Approved	Hypnotic, antimigraine agent	0.170
	Amitriptyline hydrochloride	Approved	Analgesic	0.167
	Clomipramine	Approved	Tricyclic, Antidepressive Agents	0.197
	Nortriptyline	Approved	Antidepressant, antiasthmatic agent	0.234
	Trimipramine	Approved	Tricyclic, Antidepressive Agents	0.227

### 3.8 The Effect of DDIT4 and PFKFB4 on the Proliferation and Migration of Chondrocytes

The bioinformatics analyses have demonstrated that PFKFB4 and DDIT4 can be utilized as diagnostic markers for KOA. Moreover, these analyses have also confirmed that PFKFB4 and DDIT4 are significantly down‐regulated in osteoarthritic cartilage, which suggests that they may play a role in protecting chondrocytes. Accordingly, further research is required to determine whether PFKFB4 and DDIT4 represent potential therapeutic targets for KOA.

**FIGURE 9:**
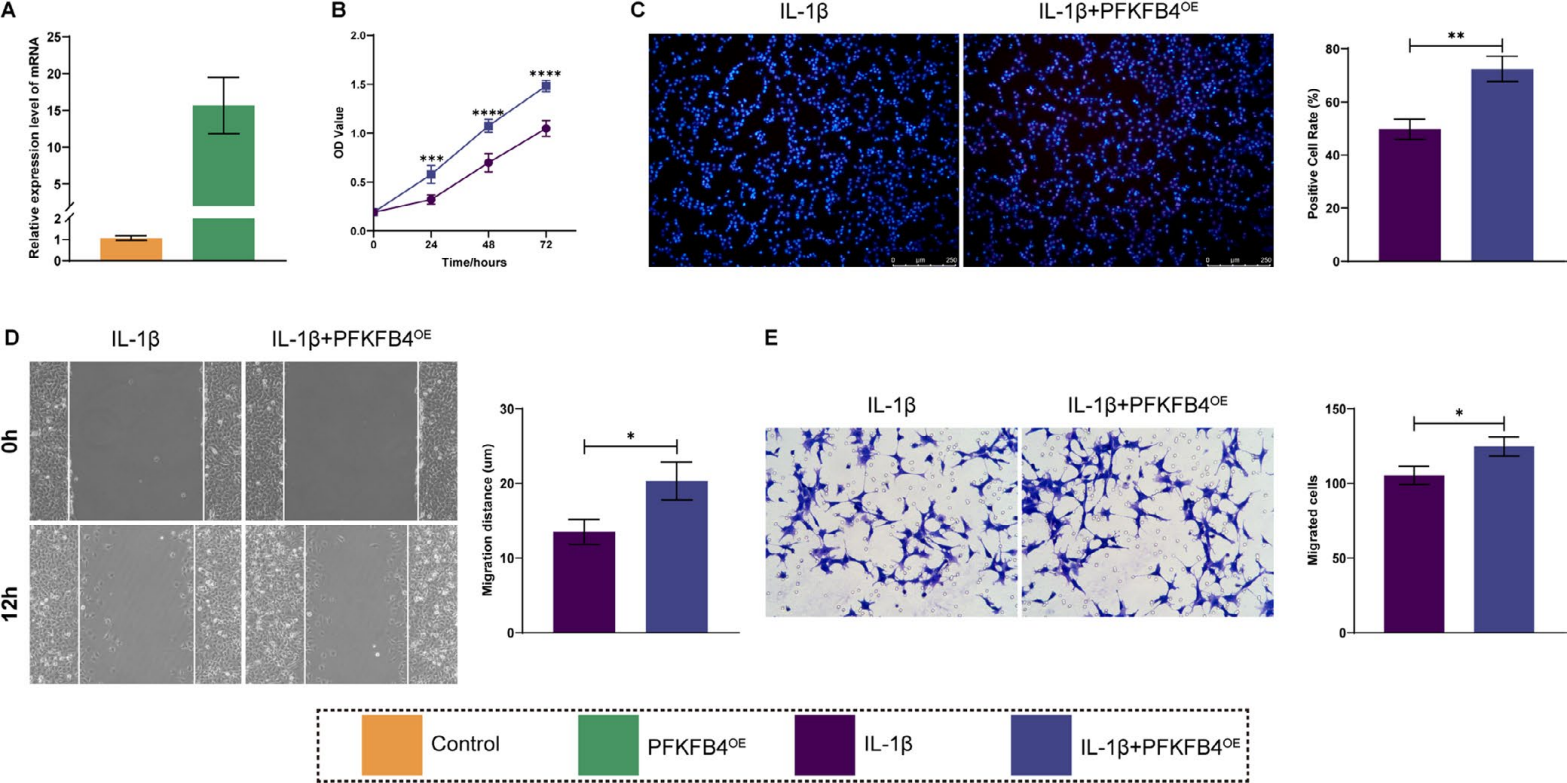
Effects on proliferation and migration of PFKFB4 overexpression were observed in vitro. (A) RT‐PCR was used to detect the overexpression efficiency of PFKFB4. (B, C) CCK‐8 and EDU staining were used to measure the proliferation activity. (D, E) Scratch wound test and Transwell were uesd to assay the migration ability.

**FIGURE 10:**
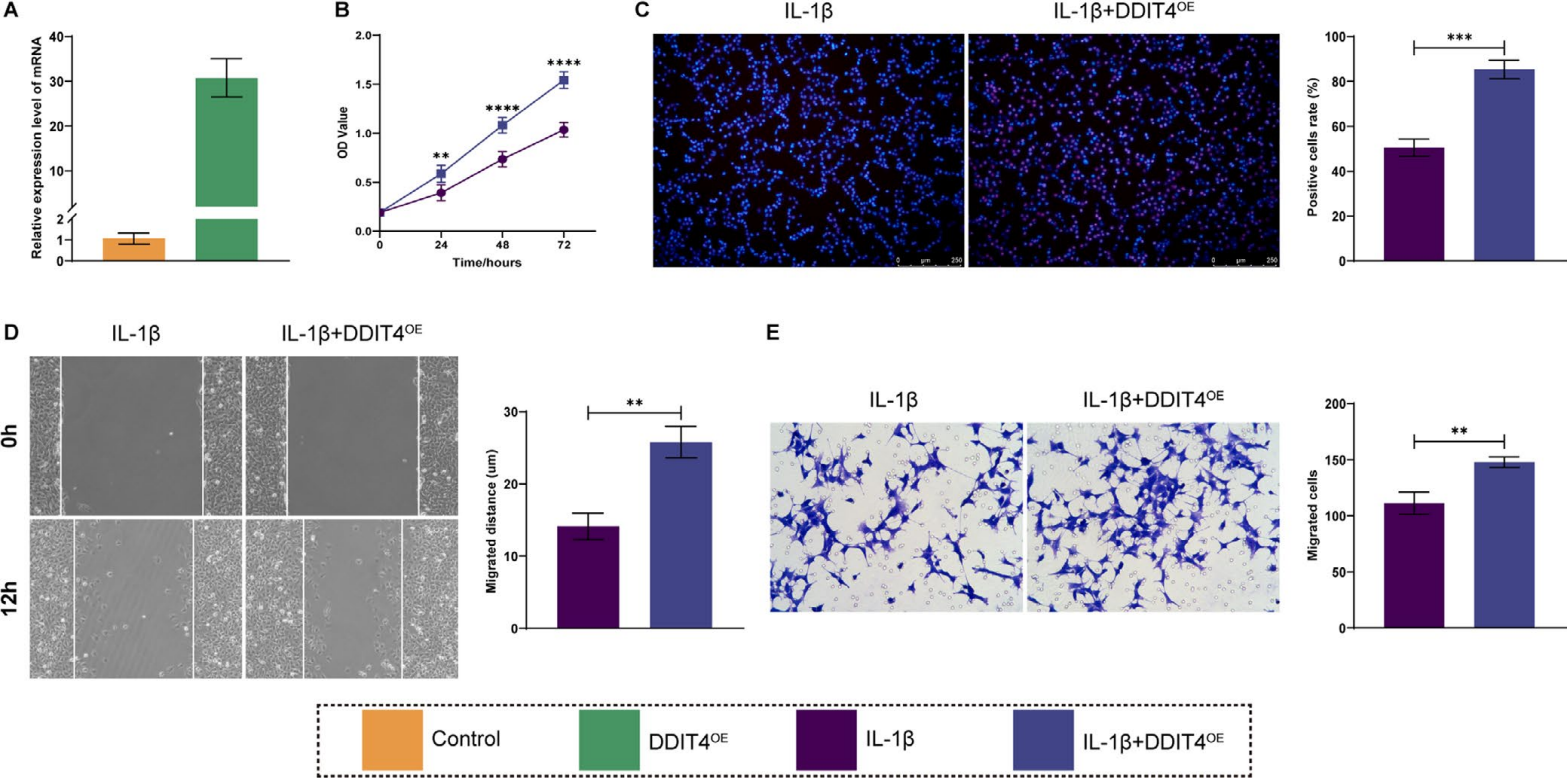
Effects on proliferation and migration of DDIT4 overexpression were observed in vitro. (A) RT‐PCR was used to detect the overexpression efficiency of DDIT4. (B, C) CCK‐8 and EDU staining were used to measure the proliferation activity. (D, E) Scratch wound test and Transwell were uesd to assay the migration ability.

Given that chondrocytes in osteoarthritis often exhibit decreased proliferation and migration, we conducted in vitro experiments to evaluate the effects of PFKFB4 and DDIT4on ATDC5 cells proliferation and migration. Specifically, IL‐1β was used to stimulate ATDC5 cells, thereby emulating the authentic state of chondrocytes in osteoarthritis. The ensuing alterations in cellular function were then evaluated in ATDC5 cells, as well as in ATDC5 cells that had been stably overexpressing PFKFB4 and DDIT4, respectively. RT‐qPCR demonstrated a notable elevation in PFKFB4 and DDIT4expression levels in the stably transformed cell lines (Figures 9A and 10A). Subsequently, as demonstrated by CCK‐8 and EDU staining, IL‐1β treatment markedly diminished the proliferative capacity relative to the control group. Conversely, overexpression of PFKFB4 and DDIT4 partially reversed this trend, as evidenced by an increase in the OD value and EDU‐positive cells (Figures 9B,C and 10B,C). Similarly, scratch wound healing and transwell assays indicated that IL‐1β treatment significantly diminished the migratory capacity. This effect was partially counteracted by overexpression of PFKFB4 and DDIT4, as evidenced by an increase in the migratory distance and number (Figures 9D,E and 10D,E).

## 4 Discussion

In this study, we screened for DE‐ARGs_CIRGs in KOA, utilizing MR to identify candidate genes with significant causal relationships. ROC curves and boxplots were used to assess the diagnostic ability and expression level of biomarkers, respectively. Notably, among the DE‐ARGs_CIRGs, DDIT4 and PFKFB4 were found to be closely associated with KOA onset. Then, a ceRNA network was constructed for these biomarkers, and we investigated immune infiltration and potential therapeutic agents. The ceRNA network comprised 2 mRNAs, 13 miRNAs, and 11 lncRNAs, with significant infiltration of immune cells, including M2 macrophages, resting dendritic cells, and resting mast cells. Additionally, four drugs—estradiol, bisphenol A, nortriptyline, and trimipramine—emerged as potentially relevant for KOA treatment.

### 4.1 Relationship Between Circadian Genes and Aging‐Induced KOA

To elucidate the molecular functions and mechanisms of action of the DE‐ARGs_CIRGs in KOA, the GO and KEGG enrichment analyses were conducted. Both GO and KEGG analyses co‐enriched the circadian rhythm pathway, with GSVA also highlighting the mammalian circadian rhythm pathway, suggesting its involvement in aging‐induced KOA development. In KOA, circadian rhythm disruption may impair chondrocyte metabolism and repair capacity, accelerating cartilage degeneration [37]. One study indicated that the expression of CIRGs decreased during the early stages of cartilage degeneration in a mouse model of DMM surgery‐induced arthritis, implying that circadian rhythm may play a role in initiating the disease process [38]. Further analyses of gene expression patterns in human cartilage revealed significant suppression of the circadian rhythm pathway in KOA patients [39]. Immunohistochemical assessments demonstrated that most normal chondrocytes expressed circadian rhythm genes at high levels, while expression in KOA cartilage tissues was reduced by approximately 50%, with levels progressively decreasing with disease severity [39]. These findings further substantiate the significant role of circadian rhythms in the development of KOA.

### 4.2 Identification and Roles Validation of KOA Biomarkers

The combination of bioinformatics and MR for biomarker screening not only enhances the accuracy and reliability of biomarker identification but also offers high reproducibility and generalizability, providing new insights for early diagnosis and precision therapy. Two biomarkers, PFKFB4 and DDIT4, were identified in this study using a combined approach of bioinformatics and MR. Detection of biomarkers in clinical samples revealed reduced expression of PFKFB4 and DDIT4 in KOA cartilage, further validating the accuracy of the identification results. PFKFB4 is a key metabolic regulatory enzyme that regulates cellular energy metabolism by synthesizing fructose‐2,6‐bisphosphate, promoting glycolysis and inhibiting gluconeogenesis [40, 41]. PFKFB4 may play an important role in maintaining energy metabolism and function of chondrocytes by regulating glycolysis. In addition, PFKFB4 may also improve chondrocyte survival under inflammation and mechanical stress by regulating cellular energy status. DDIT4 (DNA damage‐induced transcription factor 4), also known as REDD1, is an important cellular stress response protein involved in regulating cellular responses to environmental stresses such as hypoxia and nutrient deficiencies [42]. DDIT4 is mainly involved in the cellular response to environmental stresses such as hypoxia and nutrient deficiencies through the inhibition of the mTOR signaling pathway, it regulates cell growth, proliferation and metabolism, and plays a key role in cell cycle, apoptosis and energy metabolism [43, 44]. The regulatory role of DDIT4 may help to maintain the metabolic homeostasis of articular chondrocytes, improve their function, and attenuate cartilage degradation. In addition, DDIT4 may play a protective role in the pathological process of KOA by enhancing cellular adaptation to stress. Currently, there are few studies on the effects of PFKFB4 and DDIT4 in KOA. In this study, we found that the overexpression of PFKFB4 and DDIT4 promotes the restoration of chondrocyte proliferation and migration abilities through in vitro experiments. However, the specific underlying mechanisms remain to be further elucidated.

### 4.3 Comprehensive Analysis of KOA Biomarkers

GSEA on these biomarkers were conducted to explore their roles and underlying mechanisms in KOA. GSEA pathway analysis of biomarkers identified several significant pathways associated with KOA, including hypoxia, TNF signaling pathways via NFKB, EMT, P53 pathway, mitotic spindle formation and oxidative phosphorylation. Hypoxia is closely linked to KOA, as it prompts cells to shift toward anaerobic glycolysis for energy production, resulting in the accumulation of metabolites such as lactic acid, which adversely affects chondrocyte function and survival [45]. Hypoxia conditions also stimulate the release of inflammatory factors, including TNF‐α and IL‐1β, further exacerbating the inflammatory response in the joints [46]. Additionally, hypoxia also induces apoptosis of chondrocytes, reducing the population of functional chondrocytes, and further contributing to cartilage degradation and the progression of KOA [47]. TNF‐α is a pivotal inflammatory cytokine, which plays a key role in the onset and progression of KOA through activation of the NF‐κB signaling pathway [48, 49]. This activation results in the translocation of NF‐κB into the nucleus, initiating the expression of apoptosis‐related genes [50]. Furthermore, activation of the NF‐κB pathway also increases the expression of matrix metalloproteinases and degradative enzymes, leading to degradation of the articular cartilage matrix [51]. EMT, characterized by the transformation of epithelial cells into cells with mesenchymal properties, is significant in various physiological and pathological processes [52, 53, 54]. In KOA, EMT facilitates the transformation of chondrocytes into mesenchymal‐like cells, contributing to cartilage destruction [55]. The p53 protein, a crucial tumor suppressor, regulates the cell cycle, DNA repair, and apoptosis [56]. In response to mechanical stress and oxidative stress, the p53 signaling pathway can activate DNA repair mechanisms to maintain chondrocyte stability and function. However, prolonged exposure to stress may lead to p53 dysfunction, impairing its ability to effectively repair DNA damage, which results in chondrocyte dysfunction [57]. Mitotic spindle formation, a microtubular structure essential during cell division, which is mainly involved in chromosome allocation, cell morphology adjustment and cell cycle regulation [58]. Normal spindle function is essential for the synthesis of extracellular matrix by chondrocytes, and dysregulation may lead to increased matrix degradation and promote the development of KOA [59]. Oxidative phosphorylation, a key component of cellular respiration occurring primarily in mitochondria, regulates cellular energy balance and metabolic state [60]. Abnormalities in oxidative phosphorylation can disrupt energy metabolism, adversely affecting normal cellular function and survival. Our analysis revealed that these biomarkers are implicated in biological processes related to KOA, suggesting that PFKFB4 and DDIT4 may serve as potential diagnostic biomarkers for this condition.

Examining the infiltration patterns of immune cells associated with KOA is crucial for enhancing the prognosis of patients suffering from this disease. To further investigate the role of immune infiltration in KOA, we performed an immune infiltration analysis. The results indicated significant increases in immune cell populations, such as M2 macrophages, resting dendritic cells, and resting mast cells, in the cartilage tissue of KOA compared with normal cartilage tissue, implying that they may play a role in KOA due to aging‐associated circadian rhythm disruption. M2 macrophages, known as alternatively activated macrophages, play crucial roles in inflammatory responses and tissue repair processes [61]. In KOA, M2 macrophages are particularly crucial as they contribute to joint inflammation and tissue repair, secreting a wide range of growth factors and cytokines essential for the regeneration of joint tissues [62]. In addition, M2 macrophages can mitigate inflammatory damage to joint tissues by modulating the immune response [63]. Dendritic cells are critical in the immune system for recognizing and presenting antigens to T cells, thereby initiating adaptive immune responses [64]. Disruptions in circadian rhythms may affect dendritic cells' ability to activate and present antigens, which could influence their immune response in KOA and related inflammatory states. Resting mast cells, essential components of the immune system located primarily in connective tissues, are also closely associated with KOA. Recent study utilizing single‐cell and spatial transcriptomic sequencing techniques has revealed that inflammatory and mast cell populations may play key roles in cartilage degeneration in KOA [65]. We identified several specific types of immune cells that play a pivotal role in KOA, which could guide future research directions.

The ceRNA network was analyzed to elucidate possible regulatory mechanisms of these biomarkers in the pathogenesis of KOA, and the findings indicate that XIST may influence the expression levels of PFKFB4 and DDIT4 by indirectly regulating hsa‐miR‐30d‐5p and hsa‐miR‐30c‐5p, respectively. Previous studies have demonstrated that XIST promotes apoptosis in osteoarthritic chondrocytes [66, 67]. In addition, both hsa‐miR‐30c‐5p and hsa‐miR‐30d‐5p belong to the miR‐30 family, which regulates gene expression and participates in a variety of biological processes by binding to target mRNAs, inhibiting their translation or promoting their degradation [68, 69]. These miRNAs may impact KOA progression by modulating chondrocyte function, inflammatory responses, and extracellular matrix metabolism. These results enhance our understanding of the regulatory mechanisms involved in the progression of KOA pathology.

The DGIDB and CTD databases were used to predict target molecules for biomarkers to discover new disease therapeutic targets and drugs. PFKFB4 was found to bind most strongly to estradiol by molecular docking and may serve as a potential drug for KOA therapy. In addition, bisphenol A, nortriptyline and trimipramine were also explored. Estradiol, a steroid hormone, enhances chondrocyte metabolic activity by promoting cartilage matrix synthesis and repair while inhibiting cartilage‐degrading enzyme release, thus slowing cartilage degeneration [70]. In addition, estradiol may also exert protective effects on KOA through immune modulation and the regulation of inflammatory factors [71]. An in vivo study demonstrated that estradiol reduces cartilage degeneration in osteoarthritis by inhibiting endoplasmic reticulum stress‐induced chondrocyte apoptosis [72]. Furthermore, a study simulating postmenopausal conditions through ovariectomy in female mice showed that estradiol treatment exhibited chondroprotective effects by alleviating symptoms associated with KOA [73]. Bisphenol A (C_15_H_16_O_2_) is considered as an endocrine disruptor associated with various health issues [74]. Nortriptyline and trimipramine modulate pain perception in the central nervous system by increasing norepinephrine and 5‐HT levels, potentially benefiting KOA pain management [75]. Additionally, these medications may improve sleep quality through antihistamine effects, which is vital for managing chronic conditions [76]. Although such pharmacological interventions have not been extensively reported within the context of KOA treatment, they theoretically hold promise for positively impacting this condition and are anticipated to be utilized for its management in future clinical settings. In conclusion, these drugs could provide new ideas for targeted therapy of KOA, further clinical studies are necessary to confirm their specific roles and mechanisms.

## 5 Strengths and Limitations

The main strength of this study lies in the application of a novel approach combining bioinformatics and MR to identify two circadian genes associated with aging in KOA. The expression and function of these core genes were validated through clinical samples and cell experiments, establishing their potential as biomarkers for the diagnosis and treatment of KOA, thus providing a theoretical foundation for clinical diagnosis and therapy. However, there are also some limitations. Firstly, the expression levels of these biomarkers were assessed in a small number of clinical samples. Therefore, future studies should focus on increasing the sample size to further validate the accuracy of these findings. Additionally, while the functional roles of these biomarkers were evaluated at the cellular level, the underlying mechanisms still require further targeted basic research.

## 6 Conclusion

This study successfully identified DDIT4 and PFKBF4 as potential diagnostic and therapeutic biomarkers associated with aging and circadian rhythms in KOA. These findings not only provide a theoretical basis for early diagnosis and precision treatment of the disease, but also offer new insights and directions for future research and clinical therapies.

## Author Contributions

H.L.: writing – original draft, methodology, investigation, validation, software, visualization, formal analysis. Y.Y.: writing – original draft, investigation, software, formal analysis. B.L.: validation, methodology, formal analysis. J.Y.: validation, formal analysis. P.L.: validation, formal analysis. Y.G.: writing – review and editing, resources, funding acquisition. M.Z.: writing – review and editing, supervision, project administration, funding acquisition. G.N.: writing – review and editing, supervision, resources, project administration, conceptualization.

## Ethics Statement

This study was performed in line with the principles of the Declaration of Helsinki. Approval was granted by the Ethics Committee of the Second Hospital of Shanxi Medical University (Approval Number: 2024YX‐368).

## Consent

The participants provided their written informed consent to participate in this study.

## Conflicts of Interest

The authors declare no conflicts of interest.

## Supporting information


**Figure S1.** Funnel plot of the causal relationship between biomarkers and KOA. (A–E) funnel plot of BCL3, CHPF2, DDIT4, PER2, and PFKFB4 with KOA, respectively. MR, Mendelian randomization.

**Table S1.** Detailed information of the cartilage donors.

**Table S2.** The primers sequences of RT‐qPCR.

**Table S3.** Screening results of SNP.

**Table S4.** Drug prediction results of PFKFB4.
