# Supplementary material for: Comprehensive Analysis Reveals the Potential Diagnostic Value of Biomarkers Associated With Aging and Circadian Rhythm in Knee Osteoarthritis
Source: Orthop Surg. 2025 Jan 23;17(3):922–38. doi: 10.1111/os.14370 (PMC11872380; doi:10.1111/os.14370)
Supplement: Supplementary file 1 — Figure S1. Funnel plot of the causal relationship between biomarkers and KOA. (A–E) funnel plot of BCL3, CHPF2, DDIT4, PER2, and PFKFB4 with KOA, respectively. MR, Mendelian randomization. [file OS-17-922-s002.docx]

**
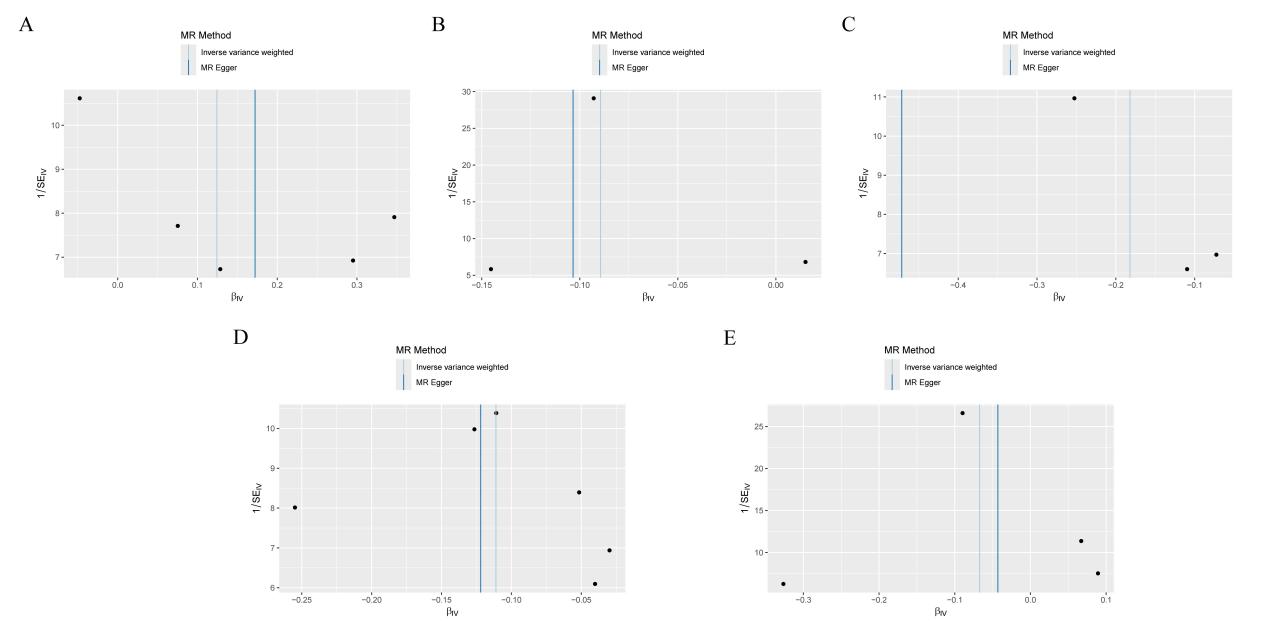
**

**Figure S1. Funnel plot of the causal relationship between biomarkers and KOA**. **A-E:** funnel plot of BCL3, CHPF2, DDIT4, PER2, and PFKFB4 with KOA, respectively. MR, Mendelian randomization.
